# A Malaysian Medical Non-Governmental Organization’s (NGO) Experience in the Emergency Response for COVID-19, Using the Whole-of-Society Collaborative Concept

**DOI:** 10.1017/dmp.2021.106

**Published:** 2021-04-06

**Authors:** Azlan Helmy Abd Samat, Aneesa Abdul Rashid, Nur Asyikin Mohd Yunus, Ahmad Munawwar Helmi Salim, Husna Musa

**Affiliations:** 1 Department of Emergency Medicine, Faculty of Medicine, Universiti Kebangsaan Malaysia Medical Centre, Jalan Yaacob Latif, Kuala Lumpur, Malaysia; 2 Islamic Medical Association of Malaysia (IMAM) Response and Relief Team (IMARET), Wilayah Persekutuan Kuala Lumpur, Malaysia; 3 Department of Family Medicine, Faculty of Medicine and Health Sciences, Universiti Putra Malaysia, Serdang, Selangor, Malaysia; 4 Malaysian Research Institute on Ageing, Universiti Putra Malaysia, Serdang, Selangor, Malaysia; 5 Department of Internal Medicine, Hospital Tengku Ampuan Rahimah, Klang, Selangor, Malaysia; 6 National Sports Medicine Centre, National Sports Institute of Malaysia, Kompleks Sukan Negara, Kuala Lumpur, Malaysia; 7 Department of Paediatrics, Faculty of Medicine and Health Sciences, Universiti Putra Malaysia, Serdang, Selangor, Malaysia

**Keywords:** coronavirus, COVID-19, intersectoral collaboration, medical societies, pandemic

## Abstract

Non-governmental organizations (NGOs) are one of the important players during a pandemic, including the Islamic Medical Association of Malaysia (IMAM) Response and Relief Team (IMARET). During the coronavirus disease (COVID-19) pandemic, IMARET played a key role in assisting health relief efforts in Malaysia. We are sharing this experience as a medical NGO’s response to the pandemic. This report presents data from the March 18 to June 10, 2020, retrieved from IMARET’s database with approval from the Executive Committee and the IMARET COVID-19 Task Force. We report that IMARET’s task force consists of 30 people, mostly medical doctors. Supplies distributed included personal protective equipment with other medical equipment, such as portable ultrasounds and ventilators. IMARET engaged with 33 collaborators and 92 partners and funders. There were 135 volunteers with the majority being medical volunteers. IMARET raised more than RM $3 million (US $740 000) garnering support from over 40 000 donors in 85 days. In conclusion, NGOs play a significant role that effectively enhance and complement the consolidated works by the authorities and public in the effort to overcome COVID-19 challenges.

## Introduction

The threat of coronavirus disease (COVID-19) in Malaysia began in January 2020 after its first imported case from China, which was aggravated following a religious mass gathering in March.^1^

Malaysia initially had the highest confirmed COVID-19 cases in Southeast Asia, recording triple-digit cases daily.^1^ However, this decreased following a nationwide, consolidated action plan.^1^ Initially, the government implemented a modified “lockdown” termed the *Movement Control Order* (*MCO*) which was subsequently replaced by the *Recovery Movement Control Order* (*RMCO*) from June 10.^2^ Similar to others, Malaysia faced challenges, which included a shortage of workforce, personal protective equipment (PPE), and medical equipment, which were amplified as the number of cases increased.

Apart from government efforts, non-governmental organizations (NGOs) and the public were generous with fundraising and even to self-produce PPEs.^1^ This response was acknowledged by the Ministry of Health (MOH) as a pertinent component in Malaysia’s action to fight COVID-19.^3^

Medical NGOs were among the active ones, including the Islamic Medical Association of Malaysia Response and Relief Team IMARET – a relief wing of the Islamic Medical Association of Malaysia (IMAM) – experienced with collaborative health relief efforts.^4^ A team named the IMARET COVID-19 Task Force was set up in response to the pandemic, whose aim included mobilizing volunteers, providing equipment to health care facilities, and supporting vulnerable communities such as the refugees and bottom 40% (B40) households.^4^

In essence, Malaysia embraced the “whole-of-society collaborative approach” pandemic preparedness model emphasized by the World Health Organization (WHO).^5^ This article aims to share IMARET’s role in this approach during the COVID-19 acute pandemic phase.

## Narrative

### COVID-19 Task Force Structure

**Figure 1.**
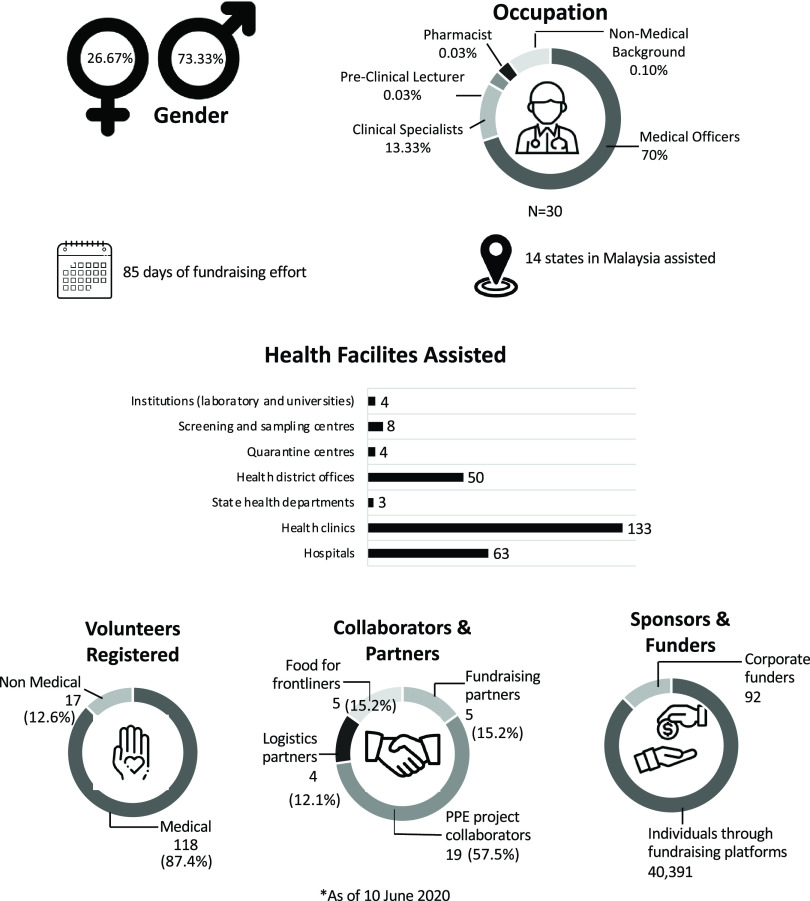
IMARET COVID-19 task force, fundraising information, and volunteers involved.

The official IMARET COVID-19 Task Force consisted of mostly males and medical officers, as shown in Figure 1. A total of 136 volunteered with IMARET, and most are from medical and allied health backgrounds. Main collaborators helped with PPE supplies, and sponsors were mostly of individuals through fundraising platforms.

### COVID-19 Task Force Response Implementation and Impact

#### Needs Assessment, Verification, and Justification

The assessment was done via feedback from professional bodies, personal contacts – and social media identified critical needs in primary care, emergency, medical, and anesthesiology. Information was then compiled, verified, and analyzed. Decisions on distributions were made based on urgency, feasibility, and cost-effectiveness.

To avoid redundancy, as well as to be synchronous with the government directives, information was also gathered from other NGOs and local health authorities.

#### Fundraising and Funds Allocation Justification

Fundraising was done via social media, focusing on critical items. The total funds collected until June 10 was RM $3 164 158.00 (US $740 067.36) (table/figure not presented). Funds allocation was made based on verified needs assessment as iterated above.

#### Procurement of Items

The IMARET COVID-19 Task Force formed a procurement team, consisting of mostly doctors who were experienced in purchasing medical equipment. The team found suppliers from China and Turkey and was responsible with ensuring the materials’ quality, suitability, the reliability of the suppliers, and logistic arrangements.

#### Logistics Arrangement

Temporary warehouses were set up with the inventory management system, and packing of items were done by volunteers. This facilitated efficient and timely distribution.

#### Delivery and Distribution

Items were delivered by volunteers following logistic planning, which included estimation of size, weight, and the delivery mode of the items for cost-effectiveness. For distribution in East Malaysia, IMARET collaborated with other NGOs and the Royal Malaysian Airforce to use mercy flights and even ferries to remote areas. When items were received by a person in charge, an acceptance letter and a delivery order letter were signed.

Table 1 shows the total distribution by IMARET throughout all 14 states in Malaysia. The highest number of PPE items distributed was 3-ply masks, followed by aprons and N95 masks. Among medical equipment, spacer/aerochambers were the most distributed items, and other items included Isopods, portable ultrasounds, and ventilators.

**Table 1.** Equipment, food, and hygiene kit distribution for refugee and bottom 40 per cent (B40) families^*^

Equipment	***n*** **(pieces/units)**
**Personal Protective Equipment (PPE)**	
Hood/head cover	13 405
Face shields	28 954
Inner scarves	860
Gloves	20 200
Aprons	111 157
Boots	70
3-ply masks	373 045
N95 masks	37 508
PPE sets (isolation gowns, boot covers, hood)	54 527
Isolation gowns	28 210
Jumpsuit/coverall	15 599
Boot/shoe covers	35 948
**Medical Equipment**	
Ventilators	5
Syringe pumps	9
Vital sign monitors	165
Isopods	2
Portable ultrasounds	10
Stethoscopes	65
Spacer/aerochambers	1242
Thermoscan	195
Infusion pumps	11
Craniotomy boxes	5
Intubation boxes	14
**Food and Hygiene Kit Distribution**	n (packs/pieces/cans)
Food packs for B40 families	1318
Food packs for refugee families	2292
Formula milk (cans)	47
Hygiene kits	30

*Notes:* ^*^Until June 10, 2020.

#### Compilation of Reports

Documentation was made on every delivery, and reports were curated for transparency purposes, which included short videos and posters in IMARET social media.

## Discussion

### IMARET’s Role During SARS-CoV-2/COVID-19

Medical NGOs are key players during a pandemic with a unique capacity to execute a community-based approach in providing information, forming joint-working groups, and developing strategies for effective response – hence bridging the gaps between knowledge and practice.^6^ During the SARS-CoV-2 pandemic, IMARET focused on coordination of fundraising and volunteering activities, providing medical supplies and medical volunteers, providing social support to vulnerable communities, complementing the government by identifying the unmet needs, and above all, networking with other NGOs to minimize redundancy – hence amplifying the impact of response.

### Identifying Needs

During this pandemic, most government health care facilities were instructed to facilitate screening and treatment of suspected COVID-19 patients. Echoing the strategies outlined by the WHO to contain the infection and reduce transmission among health care workers (HCWs)^7^ has resulted in a sudden change in nationwide standard operating procedure that disrupted the usual workflow and caused demands of items like tents and portable fans. IMARET’s needs assessment was pertinent here in ensuring appropriate funds allocations.

Health care workforce shortages were noted worldwide, and the Centers for Disease Control and Prevention recommended strategies, including communicating with local health care coalitions in identifying additional HCWs to be deployed in critical areas,^8^ which were adopted by Malaysia’s MOH, and IMARET had responded by recruiting medical volunteers for deployment.

PPE and medical equipment shortages were identified by IMARET through social media platforms, namely, Facebook, Instagram, Twitter, and networks such as WhatsApp and the mainstream media.^9^ IMARET complemented the MOH efforts by delivering donations of PPEs while awaiting supplies from the government. IMARET’s previous experience in managing a humanitarian crisis proved valuable^10^ as common logistic issues, such as trans-Atlantic transportation of equipment and lack of materials were overcome with the networks under the Federation of Islamic Medical Associations and national agencies, such as the Malaysian Armed Forces.

Support for the vulnerable communities was also prioritized as they have limited access to health care, food, and basic hygiene supply. IMARET has been recognized in Malaysia as one of the key advocates for marginalized communities’ well-being,^11^ thus, we assisted the national efforts for the contact tracing of refugees and screening by communicating with the community leaders.

### Collaborative Whole-of-Society Approach

Learning from the past, local communities have responded to the bureaucracy barriers identified in the health care system by forming a multidisciplinary task force under the National Security Council, creating new guidelines on information sharing, and improving communications.^12^ Past experiences have proven that a conventional state public health approach alone is insufficient. The health care system requires a holistic model incorporating advanced public health tools, local authorities, and non-governmental bodies.^13^ This collaborative whole-of-society approach adopted by the WHO emphasized the role of non-state players, such as the community and NGOs, assisting the government in distributing resources, obtaining and disseminating information, and representing community interest.^5^ Taiwan is an exemplary country that has embraced this concept in handling COVID-19.^13^

To play an effective role in the whole collaborative governance, an NGO requires the establishment of trust and accountability, and the ability to be resilient, flexible, and resourceful.^13^

### IMARET Crowdfunding and Social Media Utilization

Social media have been a powerful tool during this pandemic, not only for creating awareness but also for crowdfunding. IMARET launched fundraising campaigns with social media influencers and corporate companies, which received generous support throughout the 85 days of our fundraising campaign – enabling provision of PPE, medical equipment, food packs, and hygiene kits to the recipients.^14^ Hashtags such as *#kitajagakita* (“looking after one another”) and *#kitapastimenang* (“we will win”), which symbolized unity and optimism, were used to garner the public’s support.^6^

One study has shown that social media disseminate the latest news in a short time with a large geographical coverage,^15^ and it is generally acknowledged that messages with human values tend to influence more engagements with social media contents. IMARET has replicated this approach successfully through our social media contents.

### Overcoming the Challenges

IMARET received a sharp surge of funding during the early stages with overwhelming social media responses. Consequently, burn-out signs were seen among the task force. In response to this, contingency plans were formulated to systematically handle the demands by drawing an effective workflow plan for request of PPE and equipment and by emphasizing tactful messages of a medical NGO’s limitation.

Another limitation is the insufficient workforce, as most of the task force were volunteers who are medical personnel with primary duties. However, the desire to contribute and seeing social media updates played a role in motivating them. IMARET addressed the workforce issue by engaging with other NGOs and non-health-care volunteers for tasks involving logistics.

Bureaucracy, such as the protocols involved when delivering assistance to health care facilities, was also a challenge, and the anticipation of this has led us to swift rectification through our engagements with the government.

## Conclusion

NGOs are key players in the whole-of-society collaborative approach during a pandemic. This study highlights IMARET’s role during the COVID-19 pandemic in Malaysia and exemplifies how NGOs can be an effective complementary element between the authorities and communities.
